# Effect of silvopasture system on fearfulness and leg health in fast-growing broiler chickens

**DOI:** 10.1371/journal.pone.0282923

**Published:** 2023-03-23

**Authors:** Bidur Paneru, Gabriel J. Pent, Shawna Nastasi, Adam K. Downing, John F. Munsell, John H. Fike, Leonie Jacobs

**Affiliations:** 1 School of Animal Sciences, Virginia Tech, Blacksburg, Virginia, United States of America; 2 Shenandoah Valley Agricultural Research and Extension Center, Virginia Tech, Raphine, Virginia, United States of America; 3 Madison County Extension Office, Virginia Cooperative Extension, Virginia Tech, Madison, Virginia, United States of America; 4 Department of Forest Resources and Environmental Conservation, Virginia Tech, Blacksburg, Virginia, United States of America; 5 School of Plant and Environmental Sciences, Virginia Tech, Blacksburg, Virginia, United States of America; Tokat Gaziosmanpasa Universitesi, TURKEY

## Abstract

A silvopasture system intentionally integrates trees, forages, and livestock, allowing dual land use. These systems can provide high-quality habitat for broiler chickens; however, such systems have not been widely adopted by the broiler industry in the United States. The objective of this study was to examine the effect of silvopasture versus open pasture access on fearfulness and leg health in fast-growing broiler chickens. A total of 886 mixed-sex Ross 708 chicks in Experiment 1 (Exp 1) and 648 chicks in Experiment 2 (Exp 2) were housed in coops and had access to 16 (Exp 1) or 12 (Exp 2) 125m^2^ silvopasture plots (x̄ = 32% canopy cover) or open pasture plots (no canopy cover) from day 24 of age. Fearfulness was measured using a tonic immobility test (tonic immobility duration), and leg health was assessed by quantifying footpad dermatitis, hock burns, gait, and performing a latency-to-lie test on days 37–39 of age. Birds in the silvopasture treatment were less fearful than birds in the open pasture treatment. Overall, birds in both silvopasture and open pasture systems had excellent leg health. Silvopasture birds had lower footpad dermatitis scores than open pasture birds. Silvopasture birds tended to have worse gait than open pasture birds in Exp 1, but not in Exp 2. Hock burn scores and latency-to-lie did not differ between treatments in Exp 1 or Exp 2. Raising birds in silvopasture reduced fear and improved footpad health compared to birds raised in open pastures, which indicates that silvopasture systems provide some benefits for affective state and leg health in fast-growing broilers.

## Introduction

The United States (US) has three main broiler chicken production systems which mandate that birds have outdoor access. The first is certified organic production overseen by the United States Department of Agriculture (USDA), which requires that birds have access to the outdoors, with shade, shelter, exercise areas, fresh air, direct sunlight, and protection from predators year-round [1]. The second is free-range poultry production, overseen by the USDA, where producers must meet the required housing conditions to be able to sell products using the free-range label [2]. These conditions include continuous free access to the outdoors for more than 51% of the animals’ lives [2]. The third production system is pastured poultry production, which could be certified by a number of voluntary assurance schemes. Depending on those assurance schemes requirements for outdoor access can vary. Some animal-welfare benefits of these systems compared to indoor-only housing systems include more space and more opportunities to perform natural behaviors [3–6].

Broiler chicken production with outdoor access requires more land than indoor-only systems. Furthermore, birds may have negative experiences accessing the pasture when faced with extreme temperature fluctuations, presence or threat of predators, and exposure to diseases and parasites, compared to indoor-only conditions [7–9]. These conditions can increase production costs and may lead to decreased gain compared with birds in indoor housing. For instance, lower final body weights and higher feed conversion ratios were found for broilers with outdoor access compared to indoor only fast-growing birds [10]. To compensate for the additional land costs as well as potential impacts on animal performance, land productivity can be increased by utilizing a silvopasture system. A silvopasture system is an actively managed tree-forage-livestock system on a single piece of land [11, 12]. Short-term income can be generated through the production of poultry products, and long-term income can be obtained through tree or forage production. In addition, fruits and browse can have value for consumption by humans or broilers, which further helps to improve short-term economic returns by reducing food or feed costs [11]. Along with these economic benefits, silvopasture systems can reduce environmental stressors and serve as high-quality habitat for broiler chickens compared to open pasture. Yet, silvopasture systems have not been widely adopted by the broiler chicken industry.

In current production systems that allow outdoor access, ranges commonly consist of open grassland habitats [6]. Grass pastures typically do not provide birds opportunities to seek cover from aerial predators [10, 13]. Chickens prefer trees and shrubs because they provide natural cover to hide [14]. Access to pasture with trees or hedgerows can prevent predation loss, provide a milder microclimate, and improve productivity and physiological health. Olive trees or sorghum in the range resulted in no or <1% mortality due to predation for male naked-neck chickens, compared to 2.4–2.8% mortality in flocks kept in open grass pastures [15]. The microclimate in a mature-pine silvopasture system was milder compared to an open pasture system nearby in Florida, US [16]. Similarly, mean wet-bulb globe temperatures were lower in silvopasture than open pastures on the same farm in Virginia, US [17]. Access to a pasture with a mature hedgerow compared to an open pasture resulted in improved weight gain without increasing feed costs in fast-growing broilers [18].

Access to a pasture with trees rather than no or artificial shelters could potentially benefit birds’ emotional reactivity, such as fearfulness. Stadig and colleagues [19] found that far-ranging slow-growing broilers were less fearful with access to willows than close-ranging broilers that had access to artificial shelters. The complex environment of a silvopasture may reduce fearfulness similarly as indoor-only environments with enrichment objects [20, 21]. For instance, broilers or broiler breeders were less fearful when housed with perches [22], music [23], dust baths and temporary objects [21] such as balls, strings, or wall drawings [24, 25] compared to broilers without enrichments. The potential benefit for reducing fearfulness in fast-growing broilers with silvopasture access compared to open pastures has not yet been studied, even though these fast-growing broilers are the common strains used in certified-organic and some free-range and pastured poultry production systems in the US.

In conventional indoor-only broiler chicken production systems, animal welfare concerns include poor leg and foot health. In a heavy-bird (~3.7 kg) indoor-only production system in the US, 47.5% of birds had superficial or deep footpad lesions [26]. These lesions, referred to as footpad dermatitis (FPD), are problematic from an economic [27, 28] and animal welfare perspective [29]. Broilers can experience pain associated with FPD [30] and will be less motivated to access feeders and drinkers. These animals gain less weight than broilers without FPD and thus have lower carcass yields [29, 31, 32]. Providing broilers with outdoor access can reduce the prevalence of FPD. The outdoor space reduces in-house stocking density, increasing birds’ space, and thereby reducing point contamination with feces and moisture within the house. For instance, higher litter moisture levels were found in a barn system (38% vs. 28%) compared to a free-range system for turkeys between 18 and 20 weeks of age [33]. The lower litter moisture concentrations indoors reduce the chances of developing contact dermatitis, including FPD, breast dermatitis and hock burn lesions [29, 32, 34, 35]. Access to an outdoor range would therefore reduce chances of FPD and hock burn lesion development in two ways, (1) by distributing fecal material across the range (thus decreasing fecal material deposition in house) and (2) by reducing birds’ time sitting inside, which could lessen their contact with moisture and feces. A silvopasture system may encourage even greater range utilization compared to an open pasture in fast-growing broilers. For example, range use in medium-growing broilers was improved with access to conifer wigwams compared to birds in open pasture [36]. Similarly, range use in slow-growing broilers with access to short rotation coppice was 7% greater compared to birds with artificial shelters in the range [37]. In commercial operations (indoor-only, heavy-bird production) in the US, 34.5% of birds had abnormal gait or were totally lame [26]. Over 27% of indoor-only housed broilers had poor walking ability in the United Kingdom, with 3% of birds having limited ability to walk at 40 days of age [38]. Outdoor access could improve gait and reduce lameness. Slow-growing broilers that used their outdoor range had improved gait compared to birds that were indoors [39] or used the range less [19]. Yet, the potential benefit of silvopasture access for improving leg health in fast-growing broilers has not been studied.

Several studies have shown animal welfare benefits of outdoor access for fast-growing broilers [18, 40], slow-growing broilers [19, 39, 41, 42], and turkeys [33]. However, it is unclear whether the welfare benefits of pasture access, and especially silvopasture access, can be obtained for fast-growing broiler chickens, which are the most common type of broiler chicken produced in the US. Therefore, our study objective was to evaluate the effect of silvopasture versus open pasture access on fearfulness and leg health in fast-growing broiler chickens. We hypothesized that birds in silvopasture systems would be less fearful and have improved leg health (FPD, hock, gait, and latency to lie) than birds in open pasture systems.

## Materials and methods

### Animals and housing

Two experiments were conducted from April to May (Experiment 1; Exp 1) and June to August 2021 (Experiment 2; Exp 2). All procedures were approved by the Virginia Tech Institutional Animal Care and Use Committee (IACUC protocol 20–044).

In total, 886 one-day-old Ross 708 mixed-sex chicks in Exp 1 and 648 chicks in Exp 2 were obtained from a commercial hatchery (Harrisonburg, VA, USA). Birds were Marek’s vaccinated at the hatchery. Upon arrival, chicks were arbitrarily selected and housed in 12 identical pens (5.7 m^2^) with 73 or 74 birds per pen in Exp 1 and 53 or 54 birds per pen in Exp 2. Pens contained pine wood shavings (~5 cm depth), a heat lamp (day 1–8), a cardboard feed flat with feed (day 1–8), one bell drinker (Plasson® Broiler Drinker complete, Or-Akiva, Israel), and one pet champion poultry drinker (Stout Stuff LLC, China), and one feeder (Superbowl poultry feeder, LaGrange, NC, USA). The chicks were fed commercial starter (day 0–15), grower (day 15–25), and finisher (day 25–42 or 43) diets meeting requirements [43]. Ambient temperatures were 35°C on day 1 and gradually reduced to 23°C on day 22 (Exp 1) or day 23 (Exp 2). Lighting was provided continuously for the first week and reduced to 12h light and 12h dark until day 22 (Exp 1) or day 23 (Exp 2).

In Exp 1, birds from each pen were equally and randomly allocated over 16 pasture-based treatments resulting in 53 birds per plot. In Exp 2, complete pens (53–54 birds) were randomly allocated to 12 pasture-based plots with chicken coops. On day 22 (Exp 1) or day 23 (Exp 2), birds were transported for 1.5h to the pasture-based experimental site located at the Shenandoah Valley Agricultural Research and Extension Center (AREC) in Raphine, VA, US. After transportation, birds were kept inside the coops for two days (Exp 1) or one day (Exp 2) to get acclimated to their new housing conditions. From day 24, coop doors in each plot were opened at approximately 8:00 AM and closed at approximately 5:00 PM.

All pasture-based plots (125m^2^) contained a chicken coop (6.55m^2^) constructed from wood, chicken wire, and tarp [44]. Each coop contained a wooden platform perch (0.05m × 0.10m × 2.40m), feeder, and bell drinker. Coops were moved laterally across the plot each week. Plots were fenced with 1-m-high and 50-m-long FlexNet electric fences (PoultryNet®, Washington, IA, USA), connected to a 30-volt electric cattle fence. Mean pen and coop stocking densities on day 1, day 22 or 23, and day 42 or 43 for Exp 1 or Exp 2 are given in Table 1.

**Table 1:** Mean (± SEM) of pen and coop stocking density on day 1, day 22–23, and day 42–43 of age in Experiments 1 (Exp 1) and 2 (Exp 2).

Stocking Density	Pen		Coop			
	Day 1		Day 22		Day 42	
	g/m^2^	Birds/m^2^	kg/m^2^	Birds/m^2^	kg/m^2^	Birds/m^2^
**Exp 1**	518.1±0.8	13.0±0.0	7.5±0.1	8.0±0.0	20.8±0.4	7.2±0.1
	Day 1		Day 23		Day 43	
**Exp 2**	439.3±13.3	9.4±0.0	7.4±0.1	8.2±0.0	21.8±0.2	8.1±0.1

### Treatments

**Fig 1:**
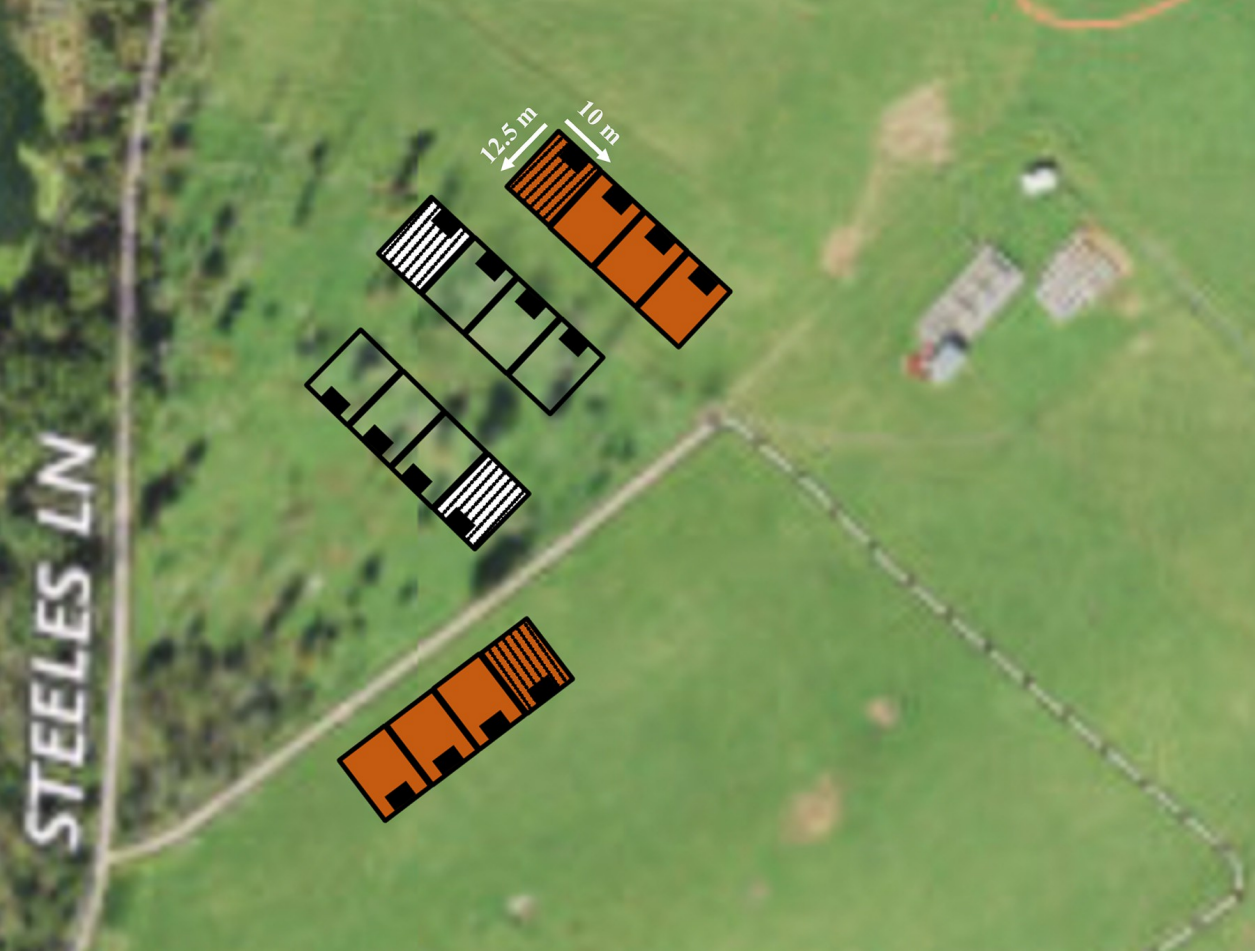
Image and illustration of experimental plots (125m^2^ each). Open grassland pasture plots (orange) and silvopasture plots (black/white) contained a chicken coop (black). Experiment 1 included 4 replicates per treatment in two locations, and Experiment 2 included 3 replicates per treatment in two locations. The omitted replicate plots for Experiment 2 are marked with a pattern fill. Map sourced from USGS National Map Viewer.

The silvopasture plots (four replicates in two locations in Exp 1, and three replicates in two locations in Exp 2; Fig 1) contained a mixed hardwood stand of black walnut (*Juglans nigrea* L.), locust (*Robinia pseudoacacia* L.) and hickory (*Carya* spp. Nutt.) trees and 30 newly planted saplings per plot (American hazelnut (*Corylus americana*), black walnut (*Juglans nigra*), persimmon (*Diospyros virginiana* L.), southern red oak (*Quercus falcata* Michx.), and southern pine (*Pinus* spp.) of approximately 30-cm height and 1-cm diameter. Saplings were planted in six rows with inter- and intra-row spacing of 1.5m. Canopy cover in all silvopasture and open pasture plots was calculated from photos using ImageJ software (1.5.3k, National Institutes of Health, Bethesda, MD, USA). The images were taken straight upwards from ground level in the center of the plot. Photos were converted to 8-bit, binarized, and then the number of black (canopy) and white (sky) pixels were calculated as a percentage of total pixels. The canopy cover for silvopasture plots was (mean±standard deviation) 31.7±16.7% in Exp 1, and 33.3±10.9% in Exp 2. Open pasture plots had no canopy cover.

The open pasture plots (four replicates in two locations in Exp 1 and three replicates in two locations in Exp 2; Fig 1) contained ground vegetation. Common herbaceous forage species in the open pasture and silvopasture plots were tall fescue (*Schedonorus arundinaceus* (Schreb.) Dumont., syn. *Lolium arundinaceum* (Schreb.) Darbysh., formerly *Festuca arundinacea* Schreb.), orchard grass (*Dactylis glomerata* L.), horse nettle (*Solanum carolinese* L.), and common milkweed (*Asclepias syriaca* L.). Forage species specific to silvopasture plots includes greenbrier (*Smilax* spp. L.), honeysuckle (*Lonicera japonica* Thunb.), Virginia creepers (*Parthenocissus quinquefolia* L. Planch.), nimble will (*Muhlenbergia schreberi* J.F. Gmel.), poison ivy (*Toxicodendron radicans* (L.) Kuntze ssp. radicans), wild basil (*Clinopodium vulgare* L.), common blue violet (*Viola sororia* Willd), and wood sorrel (*Oxalis* ssp.). Forage species specific to open pasture plots were common chickweed (*Stellaria media* (L.) Vill.), common dandelion (*Taraxacum officinale* F.H. Wigg.), and Queen Anne’s lace (*Daucus carota* L.).

### Measurements

A total of 79 or 80 birds per treatment (159 birds total) in Exp 1 and 78 birds per treatment (156 birds total) in Exp 2 received a numbered leg band on day 37 or 38 of age for individual identification. In Exp 1, 10 birds per plot were sampled; in Exp 2, 13 birds per plot were sampled. All measurements were performed on the same birds. As sampling took place on pasture, observers could not be blinded to experimental treatments.

#### Tonic immobility

Birds were tested for tonic immobility (TI) duration and TI induction on day 38 in Exp 1 and day 37 of age in Exp 2. Two observers performed the TI test in both experiments. The inter-observer agreement was tested for 10 birds and was excellent (Cronbach’s α of 0.92). The TI test was conducted as described by [21]. However, birds were tested outdoors in the pasture rather than indoors. During the TI test, the assessed birds did not have visual contact with other birds or the observer. A bird was placed on its back in a V-shaped cradle, and then TI was induced by restraining the bird with one hand on its sternum for 15 seconds while covering their head with the other hand. At the end of the induction period, both hands were gently removed. If the bird tried to right themself within 10 seconds, the induction attempt was considered failed, and the handler repeated the restraint procedure (no more than three induction attempts). In Exp 1, one bird for which TI could not be induced was replaced with another bird. In Exp 2, birds that could not be induced were included in the sample and received a TI duration of 0 seconds. After successful induction of TI, the TI duration was recorded for a maximum of 5 minutes.

#### Footpad dermatitis and hock burn lesions

Eighty birds per treatment (160 birds total) in Exp 1 and 77 or 78 birds per treatment (155 birds total) in Exp 2 were assessed for FPD and hock burn lesions on day 39 in Exp 1 and day 38 in Exp 2. FPD and hock burn lesions were scored on a 0–4 categorical scale, with increasing scores indicating worse lesions [45]. A single trained observer scored FPD and hock burn lesions in Exp 1 and Exp 2 and recorded the most severe score of a bird’s two feet or hocks.

#### Gait

Birds were evaluated individually for their walking ability and assigned a categorical gait score between 0–2, with higher scores representing worse gait [46]. A single trained observer performed the scoring by voluntarily allowing the birds to walk for at least 1.5m. If the birds did not walk, the observer stimulated the bird by gently touching their tail or vent with a rod. If the bird did not walk after gentle stimulation, the bird received the highest gait score (score 2).

#### Latency to Lie

Fifty-five or 56 birds per treatment (111 birds total) in Exp 1 and 77 birds per treatment (154 birds total) in Exp 2 were individually assessed in a latency to lie (LTL) test on days 39 or 40 in Exp 1 and days 37 or 38 in Exp 2. The test was performed as described by [47]. The LTL was recorded as an indicator of leg strength, with shorter latencies representative of poorer leg strength [47]. Individual birds were placed in an opaque plastic tub (0.93m L × 0.54m W × 0.46m H; Sterilite Corporation, Townsend, MA, USA) containing 2-3cm lukewarm (29–36°C) water for a maximum of 10 minutes. In Exp 2, the tub was covered with a barrier made from bird netting and PVC pipe to avoid escape behaviors observed in Exp 1. Four birds were tested simultaneously in separate tubs. Birds that remained standing at the end of the test received an LTL of 10 minutes. A single observer performed the tests.

#### Weather and soil conditions

Ambient temperature (°C), photosynthetically active radiation (μEm^-2^s^-1^), soil moisture (% volumetric moisture content), relative humidity (%), and dew point (°C) were measured using one Spectrum WatchDog 1000 Series MicroStation (Spectrum Technologies, Inc., Aurora, IL, USA) per treatment from day 27 to day 43 in Exp 1.

### Statistical analysis

Data of both experiments were analyzed separately in JMP Pro 16 (SAS Institute Inc., Cary, NC, USA). Data residuals of continuous response variables were assessed for the normality of their distribution by visual inspection of normal quantile plots. Data residuals of TI duration and LTL were not normally distributed. TI duration was log-transformed and analyzed using a mixed model with treatment as fixed factor and plot as random factor. TI attempts (counts) were analyzed in SAS Studio 3.8 (SAS Institute Inc., Cary, NC, USA) with a linear mixed model with a Poisson distribution, using treatment as fixed factor and plot as random factor. Transformation of LTL did not result in normality of residuals; thus, plot means were calculated to obtain a single measure per experimental unit and avoid pseudo-replication. These were analyzed in a mixed model with treatment as fixed factor and plot as random variable. Ordinal response variables (FPD, hock, and gait scores) were analyzed in SAS Studio using generalized linear mixed models with a multinomial (ordered) distribution, with treatment as fixed factor and plot as random factor. Outliers were not removed from the dataset. The threshold for significance was set at p≤0.05 and for a trend at p≤0.10. Raw means and standard errors are reported unless otherwise noted.

## Results

### Tonic immobility

**Fig 2:**
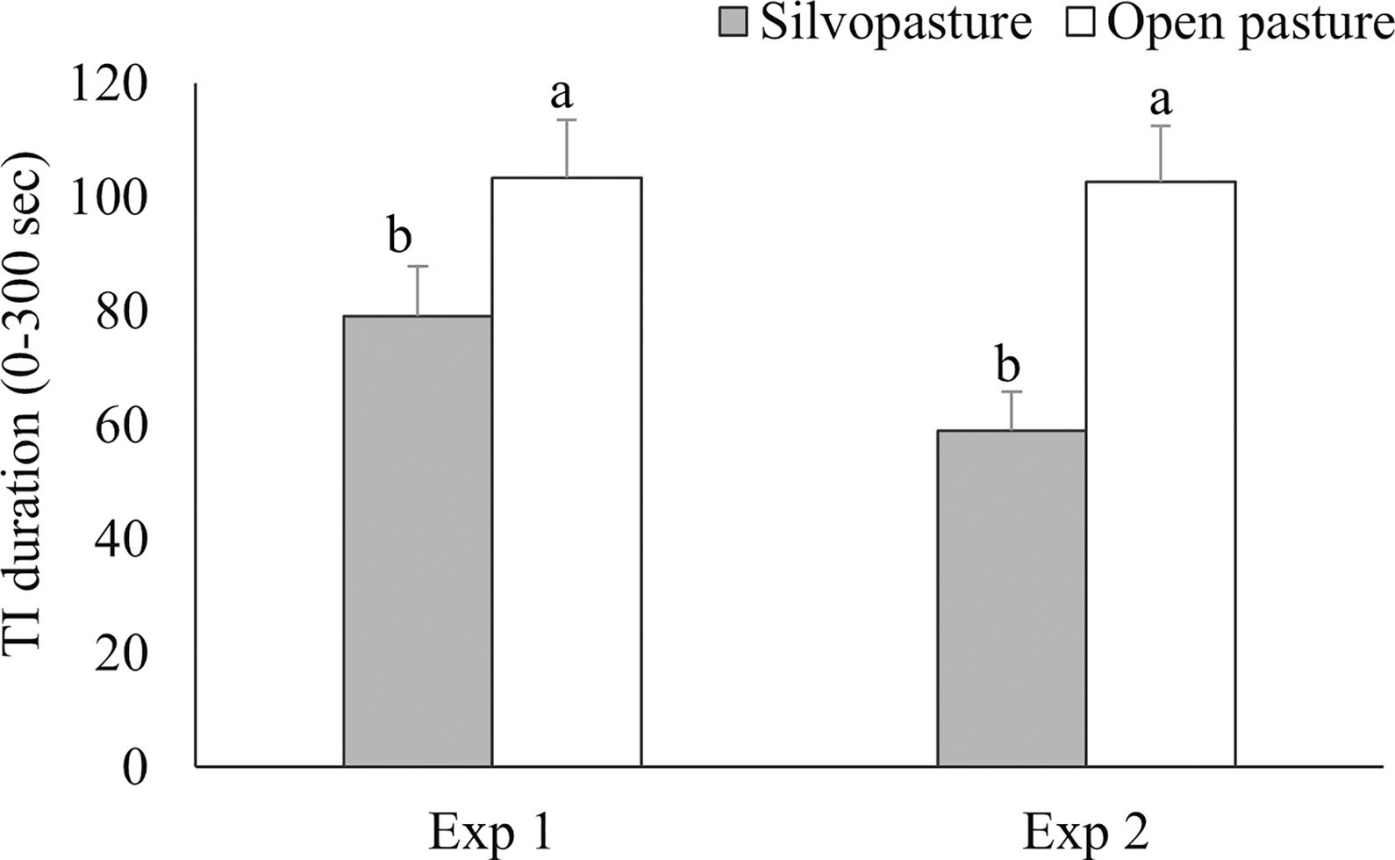
Means for tonic immobility (TI) duration (± SEM) for broilers raised in silvopasture or open pasture plots on day 38 in Experiment 1 (n = 159) and on day 37 of age in Experiment 2 (n = 156). ^AB^ Raw means within Experiment 1 with uncommon superscripts differ at p<0.10. ^ab^ Raw means within Experiment 2 with uncommon superscripts differ at p<0.05.

Silvopasture birds tended to show shorter TI durations than open pasture birds in Exp 1 (F_1,14_ = 3.89, p = 0.069) and were shorter in Exp 2 (F_1,10_ = 24.04, p<0.001; Fig 2). No difference was found in the number of attempts to induce TI among birds in the silvopasture and open pasture treatments in Exp 1 (F_1,143_ = 0.19; p = 0.664) or Exp 2 (F_1,143_ = 0.73; p = 0.395).

### Leg health

**Fig 3:**
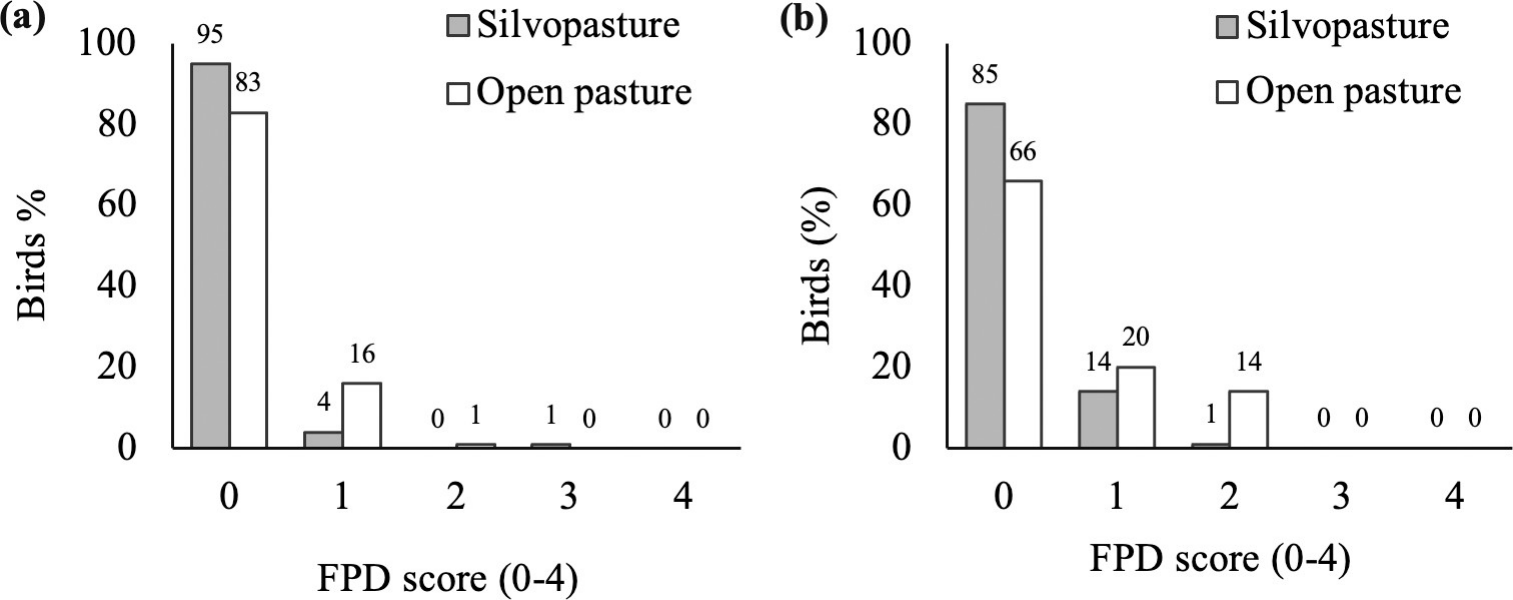
Percentage of birds with footpad dermatitis (FPD) scores 0–4 in the silvopasture and open pasture treatments. (a) On day 39 of age in Experiment 1 (n = 159). (b) On day 38 of age in Experiment 2 (n = 155).

**Fig 4:**
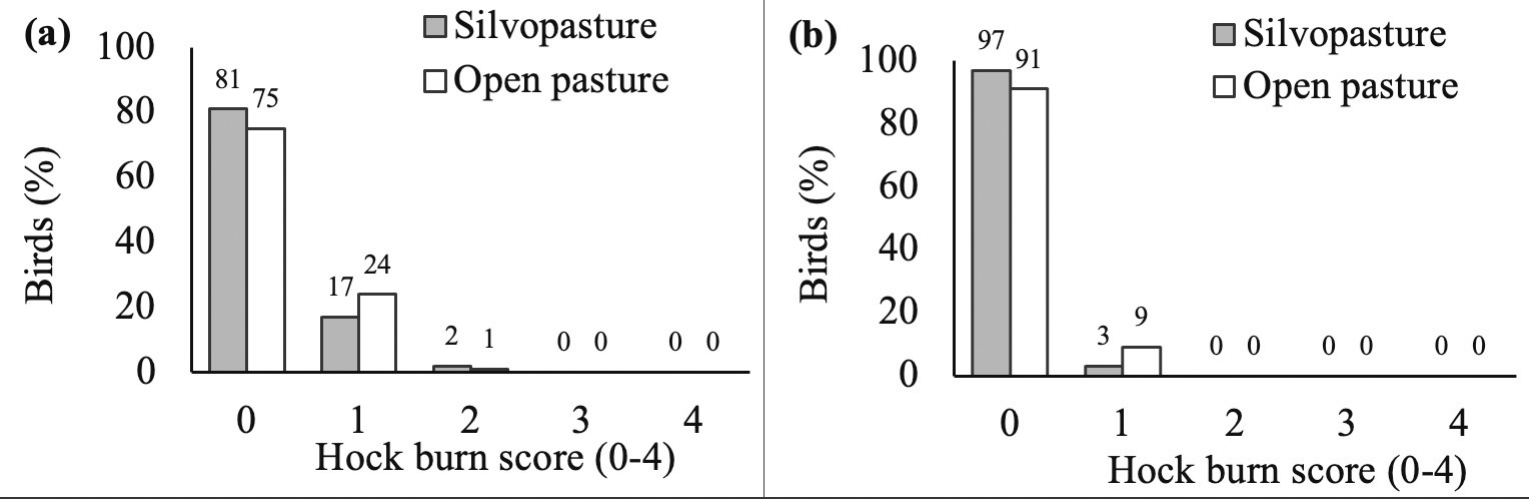
Percentage of birds with hock burn scores 0–4 in the silvopasture and open pasture treatments. (a) On day 39 of age in Experiment 1 (n = 159). (b) On day 38 of age in Experiment 2 (n = 155).

**Fig 5:**
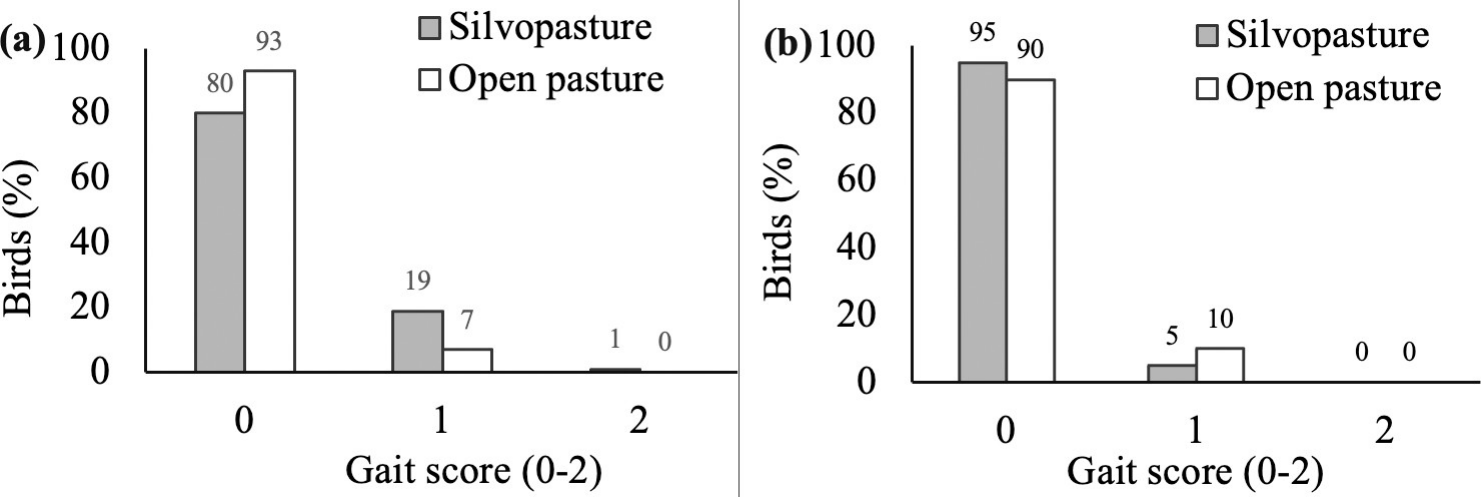
Percentage of birds with gait score 0–2 in the silvopasture and open pasture treatments. (a) On day 39 of age in Experiment 1 (n = 160). (b) On day 38 of age in Experiment 2 (n = 155).

Silvopasture birds tended to have lower (improved) FPD scores than open pasture birds in Exp 1 (F_1,141_ = 3.30; p = 0.071) and had lower scores in Exp 2 (F_1,143_ = 5.61; p = 0.019; Fig 3). Mean FPD scores were 0.08±0.04 (Exp 1) and 0.17±0.05 (Exp 2) for silvopasture birds, and 0.19±0.05 (Exp 1) and 0.48±0.08 (Exp 2) for open pasture birds. Hock burn scores did not differ between treatments in Exp 1 (F_1,142_ = 2.14; p = 0.146) or Exp 2 (F_1,143_ = 2.66; p = 0.105; Fig 4). Mean hock burn scores were 0.15±0.05 (Exp 1) and 0.03±0.02 (Exp 2) for birds in the silvopasture treatment, and 0.26±0.05 (Exp 1) and 0.09±0.03 (Exp 2) for birds in the open pasture treatment. In Exp 1 but not in Exp 2, silvopasture birds tended to have worse gait scores than open pasture birds (F_1,143_ = 2.96; p = 0.088 in Exp 1 and F_1,143_ = 1.44; p = 0.231 in Exp 2). The majority of sampled birds showed excellent gait, with ≥80% of birds having a gait score 0 (Fig 5). Mean gait scores were 0.21±0.05 (Exp 1) and 0.05±0.03 (Exp 2) for silvopasture birds, and 0.08±0.03 (Exp 1) and 0.10±0.04 (Exp 2) for open pasture birds. No difference was found in LTL between silvopasture birds and open pasture birds in Exp 1 (F_1,14_ = 1.04, p = 0.324) and Exp 2 (F_1,10_ = 1.22, p = 0.295). The mean LTL was 452±28 sec (Exp 1) and 523±19 sec (Exp 2) for birds in silvopasture, and 403±28 sec (Exp 1) and 549±13 sec (Exp 2) for birds in open pasture.

### Weather and soil conditions

Ambient temperature, photosynthetically active radiation, soil moisture, relative humidity, and dew point in the silvopasture and open pasture treatments in Exp 1 are presented in Table 2. Although not statistically analyzed, all but one (soil moisture) values were lower in the silvopasture treatment compared to the open pasture treatment.

**Table 2:** Raw means (± SEM) of weather and soil conditions in the silvopasture and open pasture treatments from day 27 to day 43 in Experiment 1 (n = 2).

Weather and soil conditions	Silvopasture	Open pasture
**Ambient temperature (°C)**	14.1 ± 0.1	15.0 ± 0.2
**Photosynthetically active radiation (μEm**^**-2**^**s**^**-1**^)	171.4 ± 4.6	272.4 ± 6.7
**Soil moisture (% volumetric moisture content)**	99.8 ± 0.1	16.8 ± 0.2
**Relative humidity (%)**	64.8 ± 0.3	70.9 ± 0.2
**Dew point (°C)**	6.5 ± 0.1	8.0 ± 0.1

## Discussion

This study investigated the effect of silvopasture versus open pasture access on fearfulness using a TI test and on leg health using FPD scores, hock burn scores, gait scores, and LTL in fast-growing broiler chickens. We found that pasture type impacted fearfulness, FPD, and tended to impact gait scores, with silvopasture access showing improvements for all measures besides gait, and no difference in hock burns or LTL.

### Tonic immobility

The shorter TI duration in birds in the silvopasture treatment in Exp 1 and Exp 2 indicates that those birds were less fearful than birds in the open pasture treatment [48]. TI durations in the current study were shorter than durations reported in previous research with conventional broilers raised in different housing conditions; indoor-only [10, 42, 49, 50], indoor housing with outdoor access [10, 49–51], and indoor housing with environmental enrichment [10, 24, 52–54]. TI durations (103–104 sec) in our open pasture treatments were comparable to the duration of a slow-growing broiler strain housed with outdoor access (108 sec; [42]). Far-ranging slow-growing broilers were less fearful with access to willows than close-ranging broilers with access to artificial shelters [19], suggesting that trees in the range were more meaningful to reduce fearfulness than artificial shelters in their study. This is in line with findings in our study, as we also found birds with access to trees to be less fearful than birds without trees in the range. Lack of overhead cover in open pasture plots could have increased the birds’ chance of encountering predators, increasing broilers’ fear and anxiety [55] compared to birds in the silvopasture plots. In the current study no mortality from areal predation occurred, yet one account of predation by a ground predator was observed in an open pasture plot (Exp 1), thought to be caused by a raccoon (*Procyon lotor*) when birds were 42 days of age, which was 4 days after fear was assessed.

In addition to being obscured from predators, we argue that a more complex outdoor range with trees could serve a similar function as enrichments provided when housed indoors, either biologically relevant (perches) or biologically less relevant (such as music or balls), as both enrichments and access to a complex range promote the expression of natural behaviors [3, 5, 56, 57]. Broilers raised with environmental complexity in indoor-only housing conditions were less fearful compared to broilers housed in simpler conditions. For instance, Arbor Acres birds with music [23] and Ross 708 birds with dust baths, perches, and temporary objects [21] were less fearful than without enrichments. Our results indicate that allowing fast-growing broiler chickens access to a silvopasture range reduces fearfulness compared to access to an open pasture range.

### Footpad dermatitis and hock burn lesions

Broilers with access to silvopasture had better FPD scores than broilers with access to open pasture. Five percent of birds in Exp 1 and 15% of birds in Exp 2 had mild or severe FPD scores in the silvopasture treatment, which is lower or comparable to reported prevalences of 12% and 22% in slow-growing broilers with access to olive trees or sorghum [41], and lower than prevalences of 68% and 72% in slow-growing broilers with access to willows or artificial shelters [19]. Litter (soil) moisture content is identified as a key risk factor for the development of contact dermatitis [32, 35, 58]. The lower FPD prevalence and severity in silvopasture plots, even with higher soil moisture content (Table 2), may be due to increased range use in silvopasture plots compared to open pasture plots, which could have reduced the in-coop stocking density, reduced fecal material within the coop, and reduced birds’ time sitting inside. Despite the soil moisture content being higher in the silvopasture treatment compared to the open pasture treatment, our findings show that FPD prevalence and severity in fast-growing broilers are minor in either pasture system, but especially minimal in the silvopasture system. Although not statistically assessed, FPD scores were lower in Exp 1 compared to Exp 2. Lower maximum in-coop stocking density in Exp 1 versus 2 (Table 1) could have contributed to that difference. Seasonal variation in FPD scores in fast-growing indoor-only broilers was reported, with the best outcomes during the warmest months [59], yet those results do not align with our findings, as FPD was slightly worse in summer compared to spring.

Hock health was excellent in birds with access to either open pasture or silvopasture and did not differ between treatments. Similar to FPD scores, severity and prevalence of hock burns were low in both treatments. Although no previous work has investigated hock burn lesions in fast-growing broilers with pasture access, our findings generally do align with earlier research on slow-growing broilers with outdoor access [40, 42]. Hock burn scores for silvopasture and open pasture birds in the current study were better when scored on a 5-point scale (mean scores between 0.03–0.26) than reported for slow-growing broilers from seven organic farms with outdoor access scored using a similar 4-point scale (mean score of 0.30; [42]). Birds in the silvopasture treatment had comparable or higher hock burn scores as slow-growing broilers from 25 farms with outdoor access (score 0 = 96% and score 1 = 4%; [40]), while birds in the open pasture treatment showed more severe hock burn lesions compared to those reported by [40]. These results met our expectations as FPD and hock burn lesions can show a positive association and share some of the same etiology [60].

### Gait

Most birds in the current study had excellent gait, with the majority showing no gait imperfection in either treatment group. Outdoor access has been associated with better gait in fast-growing [51, 61] and slow-growing broilers [19, 51] compared to indoor-only housing. Although [10] found no difference in gait scores in fast-growing broilers raised indoors compared to broilers with outdoor access. Even though access to pasture resulted in good gait across treatments, gait tended to be worse in the silvopasture treatment compared to the open pasture treatment in Exp 1 only. This is contrary to our hypothesis and does not align with the outcomes for other leg health indicators that were assessed, where silvopasture resulted in an improvement or no difference. The mechanism behind the difference in gait scores in Exp 1 is unclear. The lack of a difference in Exp 2 suggest that it may not be consistently due to the pasture treatment, but may have been a result of other factors.

### Latency to Lie

The LTL test measures leg strength in broilers by recording the time it takes for birds to lie down in lukewarm water [62], relying on chickens’ aversion to sit down in water, therefore, shorter latencies reflect poorer leg strength [47]. LTL did not differ between treatments in either experiment. In the current study, birds showed comparable LTL, thus suggesting comparable leg strength to slow-growing strains (mean LTL between 403–548 sec vs. 547 sec reported by [42]). Generally, leg health is worse in fast-growing broilers compared to slow-growing strains [63]. This suggests that both treatments in the current study resulted in good leg strength in fast-growing broilers, which is a major achievement in terms of animal welfare, as leg issues are a well-recognized welfare concern for fast-growing broilers [38, 64].

## Conclusion

This study evaluated the effect of silvopasture versus open pasture systems on fearfulness and leg health in fast-growing broiler chickens. To our knowledge, this is the first study to assess the impact of a pasture system (silvopasture or open pasture) on aspects of animal welfare and health of fast-growing broiler chickens. We found that providing fast-growing broilers with access to a silvopasture system from 3 weeks of age is particularly beneficial for reducing fearfulness and improving leg health, specifically footpad condition, compared to providing access to open pasture. Even though gait was worse in silvopasture-raised broilers in one of two experiments, scores were low (thus gait was not impaired) in either treatment. Leg strength was good and comparable in both treatments.
